# Changes in the genome-wide localization pattern of Sir3 in *Saccharomyces cerevisiae* during different growth stages

**DOI:** 10.5936/csbj.201304001

**Published:** 2013-06-19

**Authors:** Shu-Yun Tung, Kuan-Wei Lee, Jia-Yang Hong, Sue-Ping Lee, Hsiao-Hsuian Shen, Gunn-Guang Liou

**Affiliations:** a Institute of Molecular Biology, Academia Sinica, Taipei 11529, Taiwan, ROC; b Institute of Molecular and Genomic Medicine, National Health Research Institutes, Miaoli 35053, Taiwan, ROC; c Graduate Institute of Basic Medical Science, China Medical University, Taichung 40402, Taiwan, ROC; † These authors contributed equally to this work

**Keywords:** ChIP on chip, heterochromatin, genome-wide, Sir3

## Abstract

In budding yeast, the Sir2, Sir3 and Sir4 proteins form SIR complexes, required for the assembly of silent heterochromatin domains, and which mediate transcription silencing at the telomeres as well as at silent mating type loci. In this study, under fluorescence microscopy, we found most Sir3-GFP expressions in the logarithmic phase cells appeared as multiple punctations as expected. However, some differences in the distribution of fluorescent signals were detected in the diauxic~early stationary phase cells. To clarify these, we then used ChIP on chip assays to investigate the genome-wide localization of Sir3. In general, Sir3 binds to all 32 telomere proximal regions, the silent mating type loci and also binds to the rDNA region. However, the genome-wide localization patterns of Sir3 are different between these two distinct growth phases. We also confirmed that Sir3 binds to a recently identified secondary binding site, PAU genes, and further identified 349 Sir3-associated cluster regions. These results provide additional support in roles for Sir3 in the modulation of gene expression during physical conditions such as diauxic~early stationary phase growing. Moreover, they imply that Sir3 may be not only involved in the formation of conventional silent heterochromatin, but also able to associate with some other chromatin regions involved in epigenetic regulation.

## Introduction

In the eukaryotic nucleus, genomic DNA is packaged into chromatin. The nucleosome is the basic unit of chromatin organization and consists of around 146 base pairs of DNA wrapped twice around an octamer core of histones (H2A, H2B, H3 and H4) [1–3]. Post-translational/epigenetic modifications of histones, in association with DNA-binding and/or with histone-binding proteins, organize the genomic DNA into euchromatin and heterochromatin states [4–6]. Euchromatin is defined as the transcriptionally active portion of the genome, whereas heterochromatin is defined as transcriptionally less-active condensed chromosome regions. Epigenetic modifications of histones, including by acetylation, ADP-ribosylation, deimination, isomerization, methylation, phosphorylation, sumoylation and ubiquitination, affect the affinity of histones for DNA and histone-binding proteins, and can modulate gene expression [4, 7–9].

The budding yeast, *Saccharomyces cerevisiae*, is a eukaryotic microorganism. Its well-known silent heterochromatic DNA domains are silent mating type loci (*HML* and *HMR*), telomeric DNA regions [5, 10] and ribosomal DNA (rDNA) tandem repeats [11–12]. The silent information regulator (Sir) proteins: Sir2, Sir3 and Sir4, together form a nucleosome-binding complex, called SIR complex [13–16]. SIR complexes are required to establish and maintain silent heterochromatin domains at telomeres and at mating type loci [17–20]. Sir2 is also involved in the stabilization of ribosomal DNA (rDNA) tandem repeats [11–12]. O-acetyl-ADP-ribose (AAR or OAADPR), generated by Sir2 family proteins during NAD-dependent deacetylation [21–25], appears to be an essential/important element of SIR complex activity. AAR has been shown to associate with silent heterochromatin domains and to display a similar genomic binding pattern to that of Sir2 [26]. It has been demonstrated to cause a change in the stoichiometry and structure of the SIR complex [27] and to increase the binding affinity of Sir3 for nucleosomes [28] *in vitro*. In addition, in the presence of purified yeast nucleosome arrays in an *in vitro* system, the three Sir proteins require coupling to AAR synthesis for the formation of SIR-nucleosome filaments [29]. Based on a number of genetic and biochemical studies, several models for silent heterochromatin formation through SIR complexes spreading along the chromatin fiber, have been proposed [10, 26–27, 30–31]. Detailed mechanisms, however, remain unclear.

Sir3 contains a bromo-adjacent homology (BAH) domain in its N-terminal region (amino acids 11 to 196) [32] and an AAA ATPase-like domain (the so-called,‘‘ATPases associated with a variety of cellular activities’’-like domain) in its C-terminal region (amino acids 532 to 834) [33]. The BAH domain is thought to mediate protein-protein interactions and is found in a number of other chromatin-associated proteins, such as Orc1, Dnmt1, Rsc1, Rsc2 and Mta1 [34–35]. In Sir3, the BAH domain has been reported to be critical for interaction with nucleosomes/chromatin and Sir3 stability [29, 36–40]. The defining feature of AAA family proteins is a structurally conserved ATPase domain, containing an ATP binding site and the ability to assemble into oligomeric rings that undergo conformational change during ATP binding and hydrolysis [33]. However, Sir3 does not appear to possess catalytic ATPase activity. Two C-terminal regions of Sir3, 623–762 amino acids (aa) and 799–910 aa, can interact with the histone tails of H3 and H4 *in vitro* [8]. Two other C-terminal regions of Sir3, 464–728 aa and 832–978 aa, have been identified for Sir3 anti-parallel dimerization [41]. The 464–728 aa fragment of Sir3 has also been identified as a Sir4 interacting domain [41–42].

It has been reported that Sir3 binds to the tail and core regions of the nucleosome *in vitro*, and preferentially interacts with nucleosomes’ hypomethylated H3K79 and hypoacetylated H4K16 [29, 43]. The AAA ATPase-like domain of Sir3 has been reported to interact with unmethylated histone H3K79 and Sir4 [44]. The Sir2/4 complex is able to increase the binding affinity of Sir3 for the nucleosome, and makes Sir3 less sensitive to hyperacetylated H4K16 [28–29, 45]. However, overexpression of Sir3 results in its association with extended chromatin domains in the absence of Sir2 and Sir4 [46].

Sir proteins are located at the periphery of the nucleus in 6-10 punctate foci by fluorescence detection/trace methods [47]. It is believed that these also reflect the location of the silent heterochromatin DNA domains. However, in addition to these silent heterochromatin regions, secondary recruitment sites for Sir3 have been recently reported [48]. Sir3 has also been reported to be able to translocate from subtelomeres to the nucleolus in ageing cells [49–52]. It has been further shown that Sir3 binds to the rDNA repeat region in mixed populations of young cells [48]. We are interested to know the genome-wide distribution of Sir3 and to know whether and how this distribution pattern dynamically changes during cell growth.

When yeast cells are inoculated in a rich liquid culture such as YEPD medium, they metabolize glucose in the medium and produce ethanol. When glucose becomes limiting, cells switch to rapid growth with the fermentation of glucose at logarithmic phase to slowly grow with the aerobic utilization of ethanol and undergo a diauxic shift. After a transit diauxic shift, cells grow slowly in the postdiauxic phase as an early stage of the stationary phase. When the ethanol is totally consumed, cells stop growing, exit the proliferating stage and enter the quiescent state as the late stationary phase, a true growth arrest state, i.e., the growth period when cell numbers are no longer increasing. Many gene expressions change during the postdiauxic phase and stationary phase [53].

In this study, we mainly focused on two distinct growth phases: the logarithmic and diauxic~early stationary phases. First, we carefully examined the localization of Sir3 by a current popularly use of fluorescence microscopy. Then, we used a genome-wide approach to analyze changes in Sir3 spreading to map the genome-wide localization patterns of Sir3 via the chromatin immunoprecipitation (ChIP) on chip assay. We found that the genome-wide localization patterns of Sir3 were different from those at these two distinct growth stages, and further analyzed our newly identified Sir3-associated cluster regions in diauxic~early stationary phase cells.

## Experimental Procedures

### Yeast strains and primers

The yeast strains used for this study were W303, DMY748 (W303, Sir3-GFP) and DMY3993 (W303, Sir3-Myc) that were kindly gifts from Dr. Danesh Moazed. These wild type Sir3, Sir3-GFP and Sir3-Myc fusion genes are located at the original chromosomal Sir3 gene locus. Table S1 provids primers for semi-quantitative PCR of ChIP DNA. The assay is expressed as fold enrichment over input and is normalized to an internal control (Actin).

### Fluorescence microscopy

Fluorescence images were obtained by a 100X Plan-Apochromat 1.4 numerical aperture lens and an electron-multiplying CCD camera (Cascade II 512; Photometrics) on an inverted Olympus IX71 wild field fluorescence microscope with a DeltaVision Core system (Applied Precision). Serial focal sections of the z-axis with a spacing of 0.2 µm were collected. Deconvolution and maximal projection of the images were performed by Softworx software (Applied Precision). The analysis of the fluorescence images were performed by UVP BioSpectrum imaging system (UVP) associated software and ZEN (Carl Zeiss) packaged software.

### Electron microscopy (EM)

The preparation method of EM samples were mainly followed the manufacturer's instructions (Leica Microsystems) and modified, see previous descriptions [54–56]. Briefly, harvested yeast cells were either fixed by 0.1% glutaraldehyde and 1% formaldehyde, neutralized by 0.1 M ammonium chloride or cryofixed by a high pressure freezer, Leica EM PACT2 (Leica Microsystems), then substituted by methanol and infiltrated by embedding LR-Gold reagent on a Leica EM AFS2 (Leica Microsystems). The ultrathin sections of embedded samples were stained with uranyl acetate and lead citrate. Samples were examined and photographs taken using either a Jeol JEM 2100F or a FEI Tecnai T12 electron microscope.

### Chromatin immunoprecipitation (ChIP) on a Chip

The assays were as described previously [26] with some modifications. Briefly, precipitated chromatin DNA fragments of Sir3-Myc were obtained through association with a Myc antibody (Upstate (currently Millipore)), then, following the NimblGen standard protocol instructions, samples were processed through the steps of post IP, ligation-mediated PCR (LM-PCR), labelling with either cy5 or cy3, hybridization with *S. scerevisiae* whole genome tiling array chip (NimbleGen), until the step of scanning the chip, and data analysis using the NimbleScan program and SignalMap software. Data had been deposited in GEO (accession #GSE39437).

## Results and Discussion

### The punctate foci of Sir3 in logarithmic and diauxic~early stationary phases

In this study, first, we monitored the growth curves of the yeast strain with a wild type Sir3, Sir3-GFP and Sir3-Myc fusion gene on chromosomes at the authentic Sir3 location, respectively. We observed all three strains appeared to have similar growth curve patterns in a standard culture of YEPD medium (Figure S1A). It was suggested that fusion Sir3 gene with GFP or Myc tag should not affect the normal growth of yeast. According to the growth curves, we then focued and chose to harvest cells of all strains grown either on an OD valued around 3 of the logarithmic phase (Log) or on an OD valued around 10 of diauxic~early stationary phase (Sta) for all further experiments.

**Figure 1:**
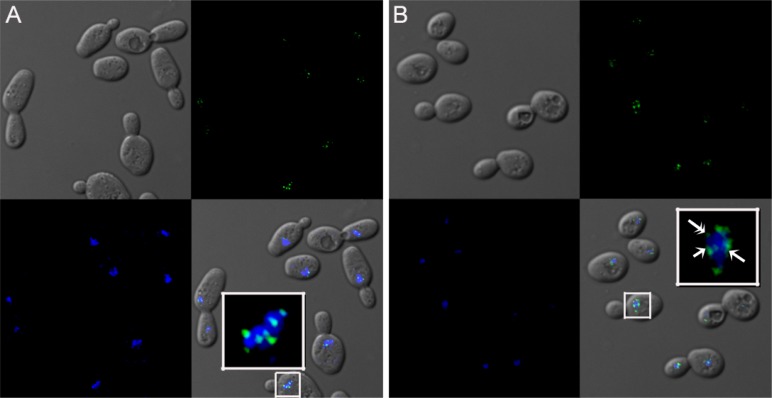
Sir3-GFP localization in cells at two different growth stages. (A) The photographs of cells: are differential interference contrast (DIC)/Nomarski image**s** (upper-left panel), GFP fluorescence image (upper-right panel), stained with DAPI (lower-left panel) and merged image (lower-right panel). The inserted white-framed box is enlarged from the small white-framed boxed area and only shows fluorescence signals. (B) is the same as (A) except cells were grown to diauxic~early stationary phase instead of taken at logarithmic phase. The weaker and smaller fluorescence signals (double-headed arrow) and the less focused fluorescence signals close to the obvious higher intensity foci (arrows) are indicated.

In Sir3-GFP strain grown to the logarithmic phase, under fluorescence microscopy, we found that Sir3 distribution appeared mainly as multiple discrete punctations (Figure. 1A, S1B) that were consistent with our expectations and other published data [8, 47, 50]. The average numbers of obvious foci per cell in two growth phase are similar (5.43 vs. 5.39 for Log vs. Sta). In addition, the distribution of the number of obvious foci per cell in two growth phases is similar (Figure S1C). However, in cells grown to the diauxic~early stationary phase, more than 85% cells (460/526 cells), we observed a similar punctate foci patterns and some less focused fluorescence close to the foci as well as numerous weaker signals (Figure 1B, S1B). Interestingly, the similar smaller and lower fluorescence signals were detected in around 2.1% (11/517) of the logarithmic phase cells. This indicates a possible association with other genomic regions and a dynamic distribution.

**Figure 2:**
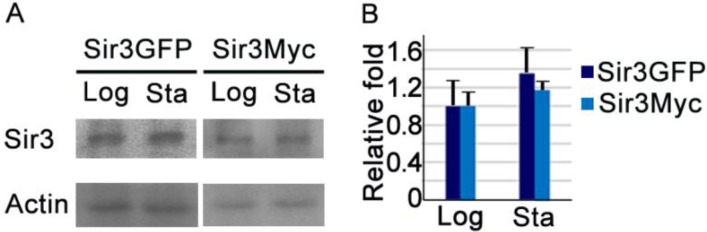
The relative amounts of Sir3 in cells at the two different growth stages. (A) Western blots of Sir3 were detected with Sir3-GFP and Sir3-Myc strains cells at the logarithmic and diauxic~early stationary phases respectively, as indicated. Actin was used as internal control and for normalization of signal. (B) The quantified results from (A) were statistically analysis and plotted as a graph. Log: logarithmic phase; Sta: diauxic~early stationary phase.

Because overexpression of Sir3 causes it to spread for a longer distance along the telomeres [46], we speculated as to whether these extra fluorescent signals were caused by a change in the amount of Sir3. Therefore we examined the amounts of Sir3 in the logarithmic and diauxic~early stationary phases. After statistical analyses, there appeared to be no significant difference (P value = 0.202) between the amounts of Sir3 in cells from these two growth stages, although there is a slightly higher amount of Sir3 in diauxic~early stationary phase cells than in the logarithmic phase cells (Figure 2). Furthermore, using Sir3-Myc strain to examine the amounts of Sir3-Myc, we also got a similar statistical results of no significant difference (P value = 0.258) (Figure 2). Moreover, the average fluorescence intensities of cell in two growth phases were also similar (Figure S1D), to indicate a similar amount of Sir3-GFP in two growth phase cells. However, in a conventional view, Sir3 should be association with silent heterochromatin regions; the extra fluorescence signals might reflect the silent heterochromatin regions. Since the heterochromatin (DNA-rich region of high electron density) and euchromatin (DNA-rich region of low electron density) regions of budding yeast cell can be distinguished on the electron microscopy (EM) image; we used the higher magnification EM images to examine whether the diauxic~early stationary phase cells exhibited obvious different heterochromatin image. Nevertheless, under electron microscopy, we saw no obvious change in the morphology of the nucleus, particularly in the heterochromatin and euchromatin regions between these two different growth stages (Figure S1E, S1F). These were implied that in the diauxic~early stationary phase, Sir3 might be able to dynamyically distribute its association locations of yeast genome at the conventional silent heterochromatin regions and also other heterochromatin regions even the euchromatin regions (because Sir3 has been reported to spread on highly transcribed genes [48]). As such, we then used a genome-wide approach to analyze the possible changes in Sir3 spreading to determine the possible meaning of the puzzling fluorescent signals of Sir3-GFP.

### The spreading of Sir3 on the conventional silent heterochromatin regions

To investigate the detailed genome-wide localization pattern of Sir3, we applied the ChIP assays in combination with a NimblGen high-resolution whole genome tiling array chip (ChIP on chip). Genome-wide localization of the Sir proteins has been previously reported using other microarray chips [57–58] and ChIP-seq [48] but without the same aims as our cases. We performed ChIP on chip experiments using ChIP of Sir3 with a Sir3-Myc-tagged gene fusion for chromosome in cells at two distinct growth stages.

**Figure 3:**
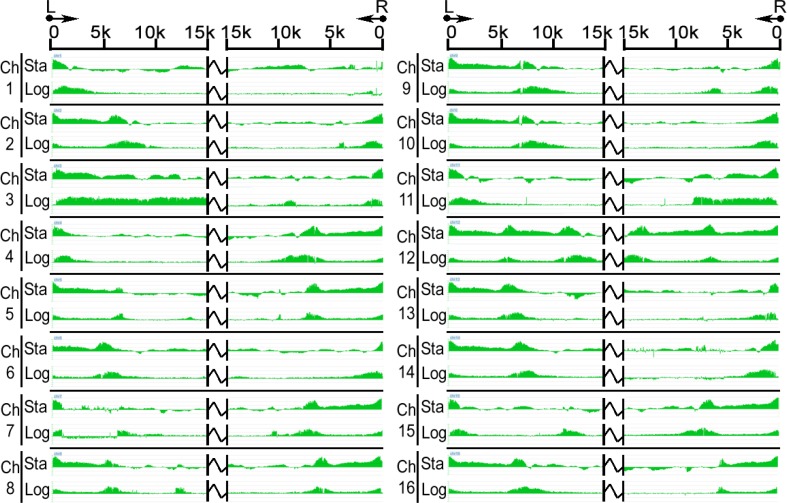
Genome-wide distribution of Sir3. Chromosomal display of Sir3 association patterns on the telomeric regions is shown. For each chromosome, the data from diauxic~early stationary phase (Sta) and logarithmic phase (Log) cells are shown on upper and lower panels, respectively, and are presented for the 15 kb region from the left (L) and right (R) chromosome ends. Chromosome number is indicated.

Using the Sir3-Myc strain grown to the logarithmic phase, we observed the expected localization of Sir3 to telomeric DNA regions (Figure 3). However, the sizes of the Sir3-associated domains varied from chromosome to chromosome and the domains were in some cases not continuous (for example, see the right arm of Chromosomes 3, 4, and 5 in Figure 3). Furthermore, at the right arm of chromosome 11, we observed an abrupt decrease in Sir3 association, suggesting the presence of a specific boundary element. We also observed a continuous domain of Sir3 associated with the left arm of chromosome 3 that extended from the telomere and encompassed the *HML* locus. In contrast, the Sir3 association domain at *HMR* was clearly separated from its telomeric association domain (Figure 3).

**Figure 4:**
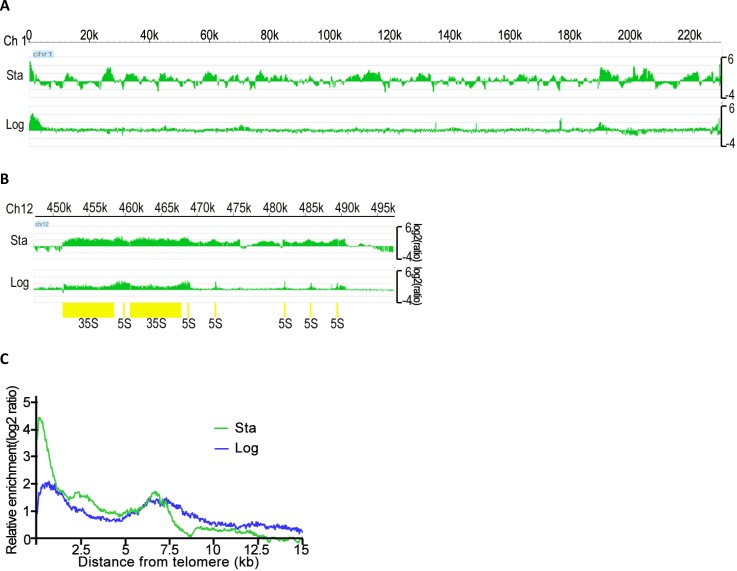
Distribution map of Sir3. (A) Different distributions of Sir3 signals in diauxic~early stationary phase (Sta) and logarithmic phase (Log) cells for the whole of Chromosome 1. Relative ratio of enrichment signal (log2) is indicated. (B) Different distributions of Sir3 signals in two growth stages (Sta & Log) cells for the rDNA region of Chromosome 12. Relative ratio of enrichment signal (log2) is indicated. (C) The moving average of Sir3 binding at all 32 yeast telomeres was plotted as a functional parameter of distance from the chromosome end. Sir3 enrichment is measured as the Log2 score of IP versus input. Data is given for Sir3 binding in diauxic~early stationary phase (Sta, green) and logarithmic phase (Log, blue).

Using same strain of cells but grown to the diauxic~early stationary phase, we observed a similar telomeric localization profile for Sir3, but a larger number of non-telomeric Sir3 peaks (Figure 4A and data not shown). In particular, we observed a broader domain of Sir3 association with ribosomal DNA repeats that extended about 20 kb to the right of the boundary edge of the region observed in logarithmic phase cells (Figure 4B). It was indicated that Sir3 was able to redistribute to the new association locations at the yeast genome in the diauxic~early stationary phase. Additionally, we used the latter condition to compare the localizations of Sir2 and Sir3 throughout the genome. We observed that Sir2 and Sir3 localized to telomeric DNA regions of all chromosomes in an almost overlapping manner (Figure S2). However, in some instances, Sir3 was associated with regions that extended further from the chromosome end than Sir2 did. These regions included the left arm of chromosome 1, the left and right arms of chromosome 5, the right arm of chromosome 12 and so on (Figure S2). These results indicate that at some telomeres, Sir3 can spread along chromatin in the apparent absence of Sir2 and provide support for previous findings suggesting that overexpression of Sir3 or Sir3 alone is sufficient for its association with extended chromatin domains and silencing [46, 59].

Furthermore, based on the concept of the binding of Sir proteins on the telomere to indicate the presence of heterochromatin, we plotted the Sir3 enrichments as a moving average function of distance from the telomere in both growth stages. We found that the relative ratio enrichment of spreading average of Sir3 on telomeres rapidly decreases to the background level at around position 9kb in diauxic~early stationary phase cells, while in the logarithmic phase cells, it decreases gradually to the basal level and extends further than position 15 kb (Figure 4C). It may be inferred that under these conditions, particularly the amounts of Sir3 were not significant different between these two growth stages (Figure 2); Sir3 appears to associate with other genomic regions resulting from a reduction in its association at telomeres and the *HM* loci when cells are growing through diauxic~early stationary phase.

### Genome-wide newly discovered localizations of Sir3

**Figure 5:**
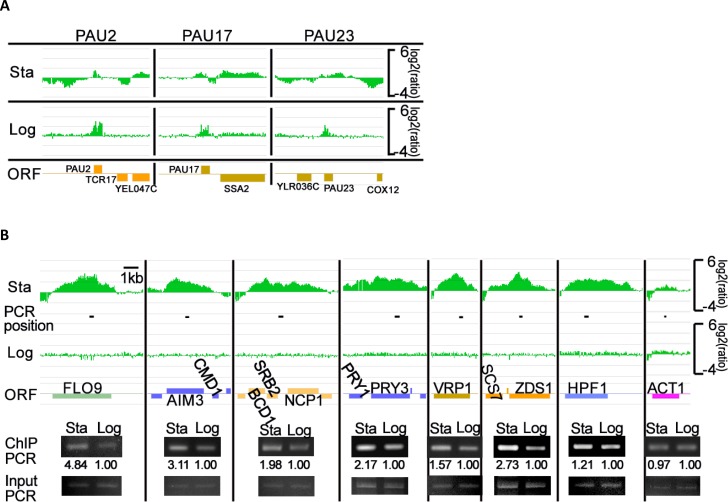
Association regions of Sir3. (A) Sir3 associates on the regions of the PAU genes at different growth phases. The different distributions of the Sir3 signal at diauxic~early stationary (Sta) and logarithmic (Log) phases, the relative ratio of Sir3 enrichment signal (log2) and the related open reading frame (ORF) are indicated. (B) Enrichment signals of Sir3 show on the newly identified regions. For each Sir3-associated cluster region, the relative enriched ratio signal of diauxic~early stationary (Sta) and logarithmic (Log) phases are shown on the top panel and the third panel from the top, respectively. The related open reading frames (ORF) (fourth panel from the top) and their positions as detected by PCR (second panel from the top) are also indicated. The ChIP PCR and input DNA PCR results are shown on the fifth panel from top and bottom panel, respectively. The quantified relative ratios of ChIP PCR list as indicated. ACT1/Actin was used as internal control and for normalization of signal.

The seripauperin (PAU) genes have been identified as secondary recruitment sites for Sir3 [48] and in this study, the Sir3 fusion protein was also detected in differing amounts, on all the PAU genes (Figure 5A). However, in diauxic~early stationary phase cells, there were many other regions associated with Sir3, on all chromosomes (Figure 4A and data not shown). After filtering out peaks with false discovery rate (FDR) using NimbleScan software, analysis revealed that aside from the known silent heterochromatin regions, there were 349 Sir3 association clusters spanning 677 genes, about 10.7% of yeast genes, and 14 intergenic regions (Table S2). We used semi-quantitative PCR to confirm some of these newly discovered Sir3 association gene cluster regions and quantified their relative ratios (Figure 5B). The results were consistent with our ChIP on chip results.

Interestingly, at most newly discovered localizations of Sir3, Sir3 were not only distributed at promoter regions but also spread on the coding regions of genes. Therefore, to classify these newly discovered localizations,, we aligned the newly identified genes by their transcription start sites (TSS) and plotted the association patterns of Sir3 binding clusters using the parameters of enrichment ratio against the distance from the TSS. Five cluster pattern sets were generated by K-means clustering analysis with K = 5 (Figure S3). The cluster 1 set was shown to have a peak of Sir3 binding at promoters (around 2kb upstream of the TSS), whereas in the cluster 2 set, the peak extended through the TSS and exhibited as a plateau. The cluster sets 3 and 5 contained one major and one minor peak. The major peak of cluster 3 appeared at upstream of TSS, but that of cluster 5 was at downstream of TSS. In addition, both the minor peaks of these two clusters were at around 2.5kb and 3kb downstream of TSS, respectively. In addition, in cluster 4 set, a broad peak was shown at around 1~2kb downstream of TSS. However, it remains to be clarified whether these 5 cluster sets individually correlate with distinct biological function/process and whether these associations of Sir3 within the coding region of genes represent a distinct functional classification and whether Sir3 plays a critical role in the modulation of gene expression in these regions.

Interestingly, some of the genes located in newly identified Sir3-associated regions were involved in either stress, aging, or were histone epigenetic modification related, for example: *TSA1, GAD1, SOD1, SGF29* and *HST1*. Nevertheless, through an ontological analysis, these genes’ products were found to be diverse with roles encompassing many biological functions, such as DNA/RNA binding, protein binding, transcriptional regulation, transferase activity and signal transduction (Table 1). At same time, other ontological analyses of biological processes and of cellular components gave similar diverse results. We also observed that these newly identified cluster regions were mostly co-localized with Sir2 associated cluster regions (data not shown). It remains to be clarified whether Sir3, or SIR complexes, play any role in the regulation of these loci.

**Table 1:** Ontological analysis of the genes located at Sir3 associated cluster regions.

GO term	Association genes frequency	Function frequency
molecular function unknown	228 out of 677 genes, 33.7%	228 out of 1993 genes, 11.4%
DNA binding	42 out of 677 genes, 6.2%	42 out of 366 genes, 11.5%
oxidoreductase activity	39 out of 677 genes, 5.8%	39 out of 276 genes, 14.1%
transmembrane transporter activity	31 out of 677 genes, 4.6%	31 out of 308 genes, 10.1%
structural molecule activity	28 out of 677 genes, 4.1%	28 out of 356 genes, 7.9%
RNA binding	28 out of 677 genes, 4.1%	28 out of 753 genes, 3.7%
ATPase activity	26 out of 677 genes, 3.8%	26 out of 230 genes, 11.3%
kinase activity	25 out of 677 genes, 3.7%	25 out of 199 genes, 12.6%
nucleic acid binding transcription factor activity	23 out of 677 genes, 3.4%	23 out of 147 genes, 15.6%
ligase activity	22 out of 677 genes, 3.2%	22 out of 181 genes, 12.2%
transferase activity, transferring acyl groups	21 out of 677 genes, 3.1%	21 out of 116 genes, 18.1%
enzyme regulator activity	21 out of 677 genes, 3.1%	21 out of 219 genes, 9.6%
protein binding transcription factor activity	17 out of 677 genes, 2.5%	17 out of 124 genes, 13.7%
mRNA binding	15 out of 677 genes, 2.2%	15 out of 68 genes, 22.1%
lyase activity	13 out of 677 genes, 1.9%	13 out of 84 genes, 15.5%
transcription factor binding	12 out of 677 genes, 1.8%	12 out of 58 genes, 20.7%
peptidase activity	11 out of 677 genes, 1.6%	11 out of 137 genes, 8.0%
methyltransferase activity	11 out of 677 genes, 1.6%	11 out of 90 genes, 12.2%
hydrolase activity, acting on carbon-nitrogen (but not peptide) bonds	11 out of 677 genes, 1.6%	11 out of 61 genes, 18.0%
phosphatase activity	11 out of 677 genes, 1.6%	11 out of 95 genes, 11.6%
small conjugating protein binding	10 out of 677 genes, 1.5%	10 out of 43 genes, 23.3%
cytoskeletal protein binding	9 out of 677 genes, 1.3%	9 out of 64 genes, 14.1%
histone binding	9 out of 677 genes, 1.3%	9 out of 39 genes, 23.1%
isomerase activity	8 out of 677 genes, 1.2%	8 out of 58 genes, 13.8%
lipid binding	8 out of 677 genes, 1.2%	8 out of 90 genes, 8.9%
transferase activity, transferring glycosyl groups	8 out of 677 genes, 1.2%	8 out of 100 genes, 8.0%
structural constituent of ribosome	7 out of 677 genes, 1.0%	7 out of 224 genes, 3.1%
protein binding, bridging	7 out of 677 genes, 1.0%	7 out of 43 genes, 16.3%
signal transducer activity	7 out of 677 genes, 1.0%	7 out of 40 genes, 17.5%
unfolded protein binding	7 out of 677 genes, 1.0%	7 out of 66 genes, 10.6%
nuclease activity	5 out of 677 genes, 0.7%	5 out of 131 genes, 3.8%
GTPase activity	5 out of 677 genes, 0.7%	5 out of 58 genes, 8.6%
enzyme binding	4 out of 677 genes, 0.6%	4 out of 49 genes, 8.2%
transferase activity, transferring alkyl or aryl (other than methyl) groups	4 out of 677 genes, 0.6%	4 out of 39 genes, 10.3%
helicase activity	4 out of 677 genes, 0.6%	4 out of 80 genes, 5.0%
protein transporter activity	4 out of 677 genes, 0.6%	4 out of 51 genes, 7.8%
translation factor activity, nucleic acid binding	3 out of 677 genes, 0.4%	3 out of 44 genes, 6.8%
hydrolase activity, acting on glycosyl bonds	3 out of 677 genes, 0.4%	3 out of 47 genes, 6.4%
ion binding	3 out of 677 genes, 0.4%	3 out of 45 genes, 6.7%
nucleotidyltransferase activity	2 out of 677 genes, 0.3%	2 out of 113 genes, 1.8%
rRNA binding	0 out of 677 genes, 0%	0 out of 92 genes, 0%
triplet codon-amino acid adaptor activity	0 out of 677 genes, 0%	0 out of 299 genes, 0%
RNA modification guide activity	0 out of 677 genes, 0%	0 out of 71 genes, 0%
other	52 out of 677 genes, 7.7%	
not_yet_annotated	2 out of 677 genes, 0.3%	

During the diauxic to stationary phase, many gene expressions undergo change [53] and Sir3 does not only spread along telomeres and HM loci, but has alternative binding sites also. According to our unpublished whole genome gene expression microarray data, compared the logarithmic phase to the diauxic~early stationary phase, among the 677 newly discovered Sir3 association genes, the transcriptions of 102 and 160 genes had been shown to be down- or up-regulated by more than 2 fold, respectively. We believe there are more Sir3 associated regions, during the period when genes dynamically change their expression in response to environmental change and its physiological effects. However, the mechanism of how Sir3 affects gene expression remains unclear. Our results imply that Sir3 might globally affect gene expression.

## Conclusion

Using advanced technologies, including newly developed hardware, operating system and analysis software, to re-examine old issues, we can sometimes uncover important new details. In this study, we started from an examination a common location of Sir3 and then got interested finding that was somewhat different from the documented record. Therefore, we applied a popular advanced technology, ChIP on chip combined with bioinformational analysis to dig for more details. In conclusion, this study provides a good example of starting from a well-known point to characterize an important new finding.

The whole genome-wide ChIP on chip strategy is able to provide a global scale view for association maps of interesting proteins. Using this approach to analyze the genome-wide association patterns of Sir3, we obtained a result that matched the conventional view when cells were in the logarithmic growth phase. However, in this study, we pointed out that the Sir3-associated domains on each telomere displayed variant sizes and some of these domains were somehow discontinuous. Moreover, some of them further exhibited an abrupt decrease of Sir3 binding to imply the possible existence of the boundary element. In addition, our result agreed with Sir3 bound to the rDNA region. Furthermore, a contrasting result when cells grew older to the diauxic~early stationary phase, allowed us to newly identify other regions of Sir3 distribution. These changes in the gene association patterns of Sir3 might relate to the modulation of the gene expressions and reflect cell responding the environmental change and starting to undergo older age processes.

Sir3 is one component of the SIR complex that is well known to be involved in the epigenetic silencing of transcription. Sir3 has already been reported to be able to associate with transcriptionally active regions, but the detail of mechanism remains unknown. Any potential duality of the role of Sir3, and whether involved in the up- or down-regulation of transcription, requires further study. The results of this study particularly highlight specific situations, such as growing into the diauxic~early stationary phase. Epigenetic effects related to Sir3 might be also involved in these biological processes. It is intriguing that Sir3 might be involved in the modulation of dynamic changes in the expression of a broad range of genes during a special physical condition. The diauxic~early stationary phase cells (OD600 = ~10 in our case) are older than cells usually used in most of bioassays. They are naturally undergoing older processes. As aging is one of the cellular functions that Sir2 is involved in, it is possible that the initial stage of aging related function of Sir3 may be directly related to its interactions with Sir2.

Overall, the genome-wide Sir3 association pattern in diauxic~early stationary phase cells has opened a new aspect in the field of epigenetics and transcriptional regulation and might provide some initial clues into the mechanisms connected with responses to environmental change and aging process.

## Supplementary Material

Changes in the genome-wide localization pattern of Sir3 in *Saccharomyces cerevisiae* during different growth stages
