# Supplementary material for: Changes in the genome-wide localization pattern of Sir3 in Saccharomyces cerevisiae during different growth stages
Source: Comput Struct Biotechnol J. 2013 Jun 19;7:e201304001. doi: 10.5936/csbj.201304001 (PMC3962127; doi:10.5936/csbj.201304001)
Supplement: Changes in the genome-wide localization pattern of Sir3 in Saccharomyces cerevisiae during different growth stages [file CSBJ-7-e201304001_SM0001.pdf]

SUPPORTING INFORMATION

Changes in the genome-wide localization pattern of Sir3 in *Saccharomyces cerevisiae* during different growth stages

Shu-Yun Tung [a,†], Kuan-Wei Lee [b,†], Jia-Yang Hong [b,†], Sue-Ping Lee [a,†], Hsiao-Hsuian Shen [b], Gunn-Guang Liou [b,c,*]

[a]*Institute of Molecular Biology, Academia Sinica, Taipei 11529, Taiwan, ROC*

[b]*Institute of Molecular and Genomic Medicine, National Health Research Institutes, Miaoli 35053, Taiwan, ROC*

[c]*Graduate Institute of Basic Medical Science, China Medical University, Taichung 40402, Taiwan, ROC*

[†]These authors contributed equally to this work

* Corresponding author. Tel.: +886 37246166 (35368); Fax: +886 37586459

E-mail address: bogun@nhri.org.tw (Gunn-Guang Liou)

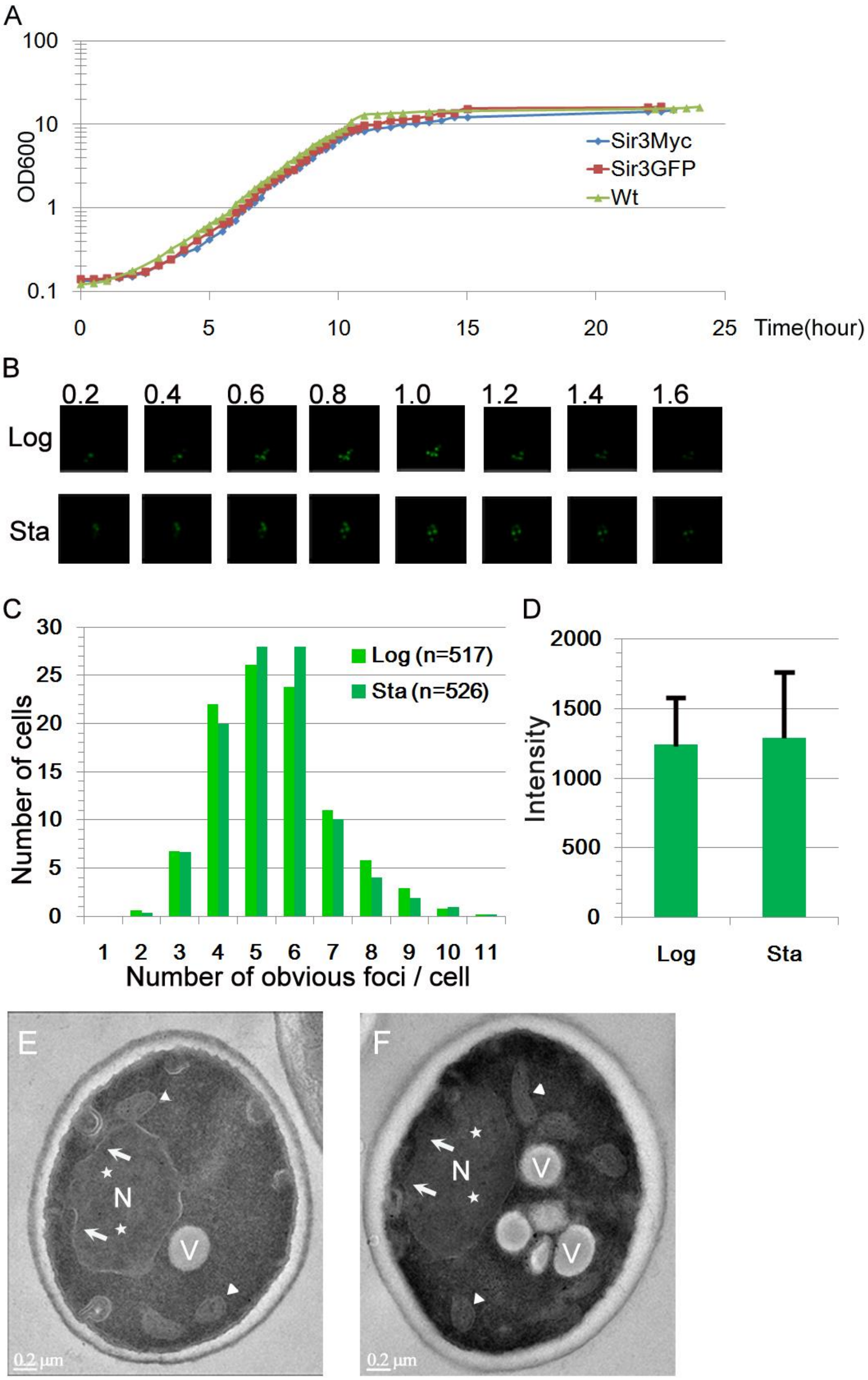

Figure S1. Yeast growth curves and its relative images (A) Growth curves of wild type Sir3 (Wt), Sir3-GFP and Sir3-Myc were recorded in a standard culture condition (YEPD medium) and then plotted as a semi-log graph. (B) The fluorescence signals of Sir3-GFP in two different growth phases. The areas of the photograph are the same as the white-framed box in Figure 1. Cells were grown to the logarithmic phase (upper panels) and diauxic~early stationary phase (bottom panels), respectively. Serial focal sections of the z-axis with spacing of 0.2 μm are shown. The position of the focal plane is indicated. (C) The distribution of number of obvious foci per cell in two different growth phases. (D) The average fluorescence intensities of cell in two different growth phases. (E) The fine structural image of a cell in the logarithmic phase by electron microscopy. (F) same as (E) except the cell grown to the diauxic~early stationary phase. N: nucleus; V: vacuole; white arrow: heterochromatin; asterisk: euchromatin; triangle arrow: mitochondria.

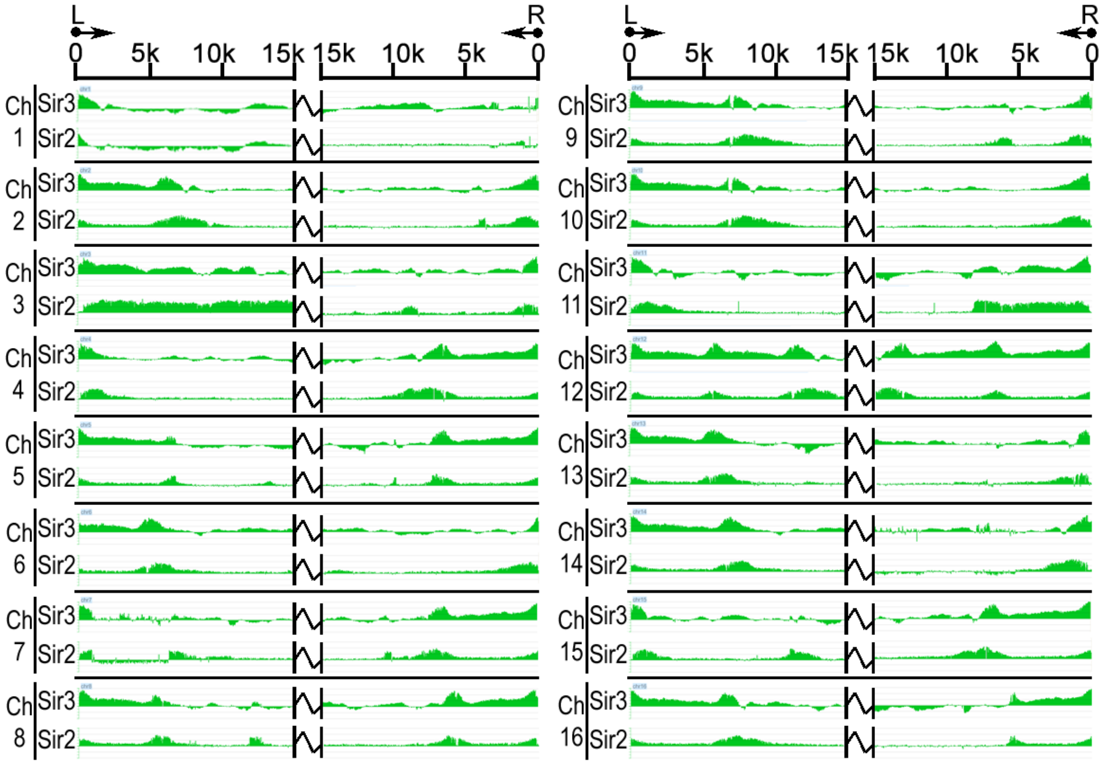

Figure S2. Chromosomal display of Sir3- and Sir2-associations at the telomeric regions. For each chromosome, Sir3 and Sir2 data are shown on upper and lower panels, respectively, and are presented for the 15 kb region from the left (L) and right (R) chromosome ends. Chromosome number is indicated.

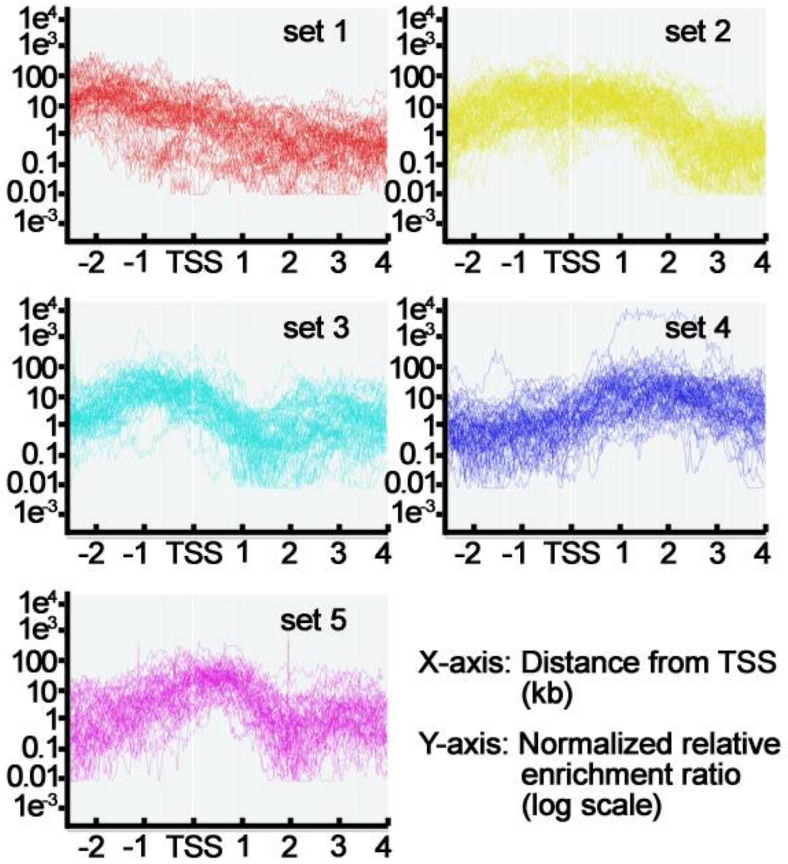

Figure S3. Five cluster pattern sets of K-means classification. The association pattern of Sir3 binding clusters at the stationary growth stage is plotted as a functional parameter of distance from the transcription start site (TSS). Relative ratios of Sir3 enrichment signals, measured as the log score of IP versus input and distance from the TSS, are indicated.
